# Modified Natural Diatomite with Sorptive and Antibacterial Properties for Respiratory Protection Applications

**DOI:** 10.3390/jfb17100484

**Published:** 2026-09-23

**Authors:** Zhanna Alsar, Aisarat Gajimuradova, Nurtai Gubaidullin, Nurzhan Serik, Fariza Zhagipar, Zinetula Insepov

**Affiliations:** 1 Private Institution “Nazarbayev University Research Administration”, Nazarbayev University, Astana 010000, Kazakhstan; zinsepov@purdue.edu; 2 Organic Agrotechnology Engineering Center, Agrotechnical Park, NCJS Saken Seifullin Kazakh Agrotechnical Research University, Astana 010011, Kazakhstan; a.gajimuradova@kazatu.edu.kz (A.G.); nur-tai.kz@mail.ru (N.G.); zhagipar.fariza@gmail.com (F.Z.); 3 JSC “Zhairem Mining and Processing Plant”, Kazzinc LLP, Karazhal 100702, Kazakhstan; nurzhan.serik1990@kazzink.com; 4 School of Nuclear Engineering, Purdue University, West Lafayette, IN 47907, USA

**Keywords:** diatomite, antibacterial properties, *Enterococcus faecium*, physicochemical modification, porous sorbent, silica-based materials, medical face masks

## Abstract

Diatomite combines a highly porous structure with strong sorption capacity, making it an attractive material for use in medical face masks. In this study, natural diatomite from the Zhalpak deposit (Mugojar Mountains, Kazakhstan) was subjected to sequential physicochemical modification to develop a sorbent for this application. This treatment sequence substantially altered the chemical composition, pore structure, and crystalline phases of the material, resulting in the modified sorbent DE-H_2_SO_4_-600f. Its interaction with Enterococcus faecium differed markedly from that of untreated diatomite: after 7 h of contact, recoverable viable bacterial counts were reduced approximately 1200-fold relative to the initial level. In a separate experiment at a high sorbent-to-liquid ratio, no bacterial growth was detected after 24 h under the tested conditions. These findings demonstrate a pronounced reduction in recoverable viable bacteria following contact with the modified diatomite and support its potential future incorporation as a functional component in medical face masks and related respiratory-protection systems.

## 1. Introduction

Diatomite, or diatomaceous earth, is a naturally occurring silica-rich material formed from the fossilized siliceous shells of diatoms. Its characteristic architecture combines high porosity and a large internal surface area with low density and chemical stability, which explainsdiatomite’s long history of use in sorption and filtration [1,2,3,4]. Deposits occur worldwide, withKazakhstan possessing substantial reserves of this mineral resource [3,5]. The combination of itsabundant reservesand intrinsically porous silica structure makes Kazakhstan diatomite an attractive basis for the development of functional sorbents.

The performance of natural diatomite is determined not only by its silica framework but also by its mineral composition and associated impurities. Natural deposits contain clay minerals, aluminosilicates, iron-containing phases, and other components that influence accessible surface area, pore structure, and surface chemistry [1,3,6]. Purification and physicochemical modification therefore provide a means of tailoring the material for a specific function. Acid treatment can remove mineral impurities and substantially modify the composition and porous structure of diatomite, while subsequent thermal treatment can induce further structural and phase transformations [1,6,7]. Recent studies further demonstrate that acid modification remains an active approach for tailoring diatomite, with sulfuric-acid treatment producing substantial changes in mineral composition, accessible surface area, pore structure, and sorption performance [8,9].

The sorption properties of diatomite extend across chemically and physically different contaminants. Natural and modified forms of diatomite have been investigated for the uptake of radionuclides and radiometals, including Th(IV), U(VI), ^60^Co(II), and ^137^Cs [10,11,12,13]. More recent studies continue to explore advanced sorbent systems for uranium and thorium removal, including surface-functionalized diatomite for U(VI), reflecting sustained interest in the development of mineral-based materials for radionuclide retention [14,15]. Thorium is of particular interest because mining and processing of thorium-bearing and rare-earth ores can introduce radionuclides into environmental and occupational exposure pathways, while adsorption on natural and modified mineral sorbents, including diatomite, provides an effective means of their retention [16]. This is relevant to respiratory protection in industrial environments where radionuclide-bearing dust or aerosol particles may be inhaled. In such applications, a sorptive layer would serve to capture and retain contaminant-bearing particles rather than to provide shielding against ionizing radiation.

The same combination of high porosity, accessible surface area, and sorption capacity is relevant to biological contaminants. Diatomite has a long history as a filtration medium, including depth filtration of microorganism-containing liquids [17,18]. Its interconnected porous structure enables particle retention not only by geometric interception but also through interactions with the internal surface of the material [17,18]. Direct adsorption of bacterial cells on diatomite carriers has also been demonstrated, and diatomaceous earth has been used as a porous matrix for bacterial immobilization [19,20]. Recent studies further show that modification of the diatomite surface can markedly influence its interaction with bacterial cells, while antibacterial diatomite-based materials have also been developed by incorporating biologically active components into the porous mineral matrix [21,22,23]. These observations show that diatomite can interact with microorganisms through mechanisms that extend beyond simple surface interception.

Medical face masks provide a particularly relevant application for this approach. Microorganisms can accumulate on worn face masks, with bacterial contamination increasing with wearing time [24]. Recent studies confirm that bacterial viability on mask surfaces remains a relevant concern during use and that the presence of biological fluids and organic components, including saliva, can influence bacterial survival [25]. Respiratory droplets interact with mask materials in ways that depend on droplet size, impact conditions, and the structure of the mask layers [26,27,28]. At the same time, current research on antibacterial face masks increasingly considers biological activity together with conventional filtration performance, emphasizing the need to combine contaminant control with the functional requirements of the mask itself [29]. Introducing a porous sorbent into the mask structure therefore offers an additional functional element: deposited liquid can be taken up by the sorbent while associated microorganisms are retained within the porous layer. Such a layer is not intended to replace the conventional filtration medium but to complement it by providing additional sorptive functionality and the potential to retain associated biological contaminants.

Natural diatomite, however, cannot simply be transferred from a mineral deposit into a functional mask material. Its composition, accessible porosity, and surface characteristics depend on both geological origin and processing. Developing a diatomite sorbent for this application therefore requires controlled purification and modification, followed by evaluation of how these treatments affect both the physicochemical characteristics of the material and its interaction with microorganisms.

Recent studies highlight several complementary directions relevant to the present work: physicochemical modification can be used to tailor the structure and sorption properties of diatomite; surface modification can alter its interaction with bacterial cells; and diatomite can serve as a matrix for antibacterial components [8,9,21,22,23]. However, the combined effect of sequential physicochemical modification of natural diatomite on both its material characteristics and its interaction with viable bacteria remains comparatively underexplored, particularly in the context of developing a sorptive component for respiratory protection.

In this study, natural diatomite from the Zhalpak deposit (Mugojar Mountains, Kazakhstan) was subjected to sequential washing and deionization, acid treatment, thermal treatment, and flux-assisted modification. Changes in chemical composition, water-extractable ionic components, pore structure, surface morphology, and crystalline phase composition were examined, and the resulting DE-H_2_SO_4_-600f sorbent was evaluated in contact with *Enterococcus faecium*. The study was undertaken to develop and characterize a modified diatomite sorbent with antibacterial properties for potential incorporation into medical face masks, while its broader sorptive characteristics may also be relevant to respiratory protection against aerosol-borne particulate contaminants.

## 2. Materials and Methods

### 2.1. Materials

Natural diatomite from the Zhalpak deposit (Mugojar Mountains, Kazakhstan) was used as the starting material. The chemical composition of the natural diatomitein this deposit, which was previously determined, is presented in Table 1. Previous characterization of the material also identified montmorillonite, kaolinite, albite, and mica [4].

Analytical-grade reagents were used throughout the purification and modification procedures. Hydrochloric acid (37%, EMSURE^®^, ACS grade; Merck, Cat. No. 100317), sulfuric acid (95–97%, EMSURE^®^, ACS grade; Merck, Darmstadt, Germany, Cat. No. 100732), and anhydrous sodium carbonate (≥99.5%, ACS reagent; Sigma-Aldrich, St. Louis, MO, USA, Cat. No. 222321) were used to prepare the required treatment solutions and for flux-assisted modification. Deionized water was used for washing, deionization, and solution preparation.

### 2.2. Preparation and Modification of Diatomite

Untreated natural diatomite was designated DE. The material was subjected to sequential purification and modification, including preliminary washing, deionization, acid treatment, thermal treatment, and flux-assisted modification. The resulting samples were designated according to the treatment applied: DE-D for deionized diatomite; DE-HCl, DE-HCl-600, and DE-HCl-900 for HCl-treated diatomite before and after calcination at 600 and 900 °C; and DE-H_2_SO_4_, DE-H_2_SO_4_-600, DE-H_2_SO_4_-900, and DE-H_2_SO_4_-600f for the corresponding H_2_SO_4_-treated samples. The numerical suffix indicates the calcination temperature, and *f* denotes the flux-treated variant.

#### 2.2.1. Preliminary Washing and Deionization

Natural diatomite rock was first crushed using a jaw crusher and subsequently ground in a drum mill. The ground material was sieved, and the fraction with a particle size <1 mm was used for further processing. This fraction was dispersed in an excess of running water and allowed to settle. The supernatant was then decanted to remove mechanical impurities, including fine sand and clay particles.

The pre-washed material was subsequently subjected to repeated washing with deionized water. The resulting purified sample was designated DE-D.

#### 2.2.2. Acid Treatment and Calcination

For hydrochloric-acid treatment, the pretreated diatomite was mixed with 5 M HCl at a diatomite-to-acid ratio of 1:5 (*w*/*v*). The suspension was maintained at 70 °C for 1.5 h under continuous stirring. After cooling and settling, the solid phase was filtered and washed with deionized water until the wash water reached neutral pH. The material was then subjected to ultrasonic treatment at 400 W and 20 °C for 3–5 min, filtered, and dried at 105 °C. The resulting material was designated DE-HCl. Portions of DE-HCl were subsequently calcined at 600 and 900 °C to obtain DE-HCl-600 and DE-HCl-900, respectively.

Sulfuric-acid treatment followed the same general procedure and diatomite-to-acid ratio (1:5, *w*/*v*), using 2 M H_2_SO_4_ as the treatment solution. After washing to neutral pH, ultrasonic treatment, filtration, and drying at 105 °C, the resulting material was designated DE-H_2_SO_4_. Portions of DE-H_2_SO_4_ were calcined at 600 and 900 °C to obtain DE-H_2_SO_4_-600 and DE-H_2_SO_4_-900, respectively.

For all calcination treatments, the samples were heated in air at a rate of 5–10 °C/min to the target temperature, held isothermally for 3 h, and then allowed to cool naturally in the furnace.

#### 2.2.3. Flux-Assisted Modification

For flux-assisted modification, Na_2_CO_3_ was added at 3 wt.% relative to the mass of the sulfuric-acid-treated diatomite during the calcination stage. The sulfuric-acid-treated material was calcined with Na_2_CO_3_ at 600 °C, and the resulting modified sorbent was designated DE-H_2_SO_4_-600f. This material was subsequently subjected to physicochemical characterization and biological evaluation.

### 2.3. Physicochemical Characterization

Physicochemical characterization of the untreated and modified diatomite samples was performed at the Core Facility of Nazarbayev University (Astana, Kazakhstan). Complementary analytical methods were used to characterize changes in chemical composition, pore structure, surface morphology, and crystalline phase composition introduced during successive stages of purification and modification.

#### 2.3.1. X-Ray Fluorescence Analysis

The chemical composition of the untreated and modified diatomite samples was determined by X-ray fluorescence spectroscopy using a PANalytical Axios Max XRF spectrometer (PANalytical B.V., Almelo, The Netherlands). Prior to analysis, the samples were ground, homogenized, and prepared as pressed pellets without the addition of a flux. Quantitative measurements were performed in five independent determinations (*n* = 5), and the results are reported as mean ± standard deviation (SD).

#### 2.3.2. Pore-Structure Analysis

The pore characteristics of the diatomite samples were examined using complementary mercury intrusion porosimetry and nitrogen gas adsorption.

Mercury intrusion porosimetry was performed using a POREMASTER 60 mercury intrusion porosimeter (Anton Paar QuantaTec Inc., Boynton Beach, FL, USA). The measurements were used to determine the pore-size distribution and accessible pore volume from the relationship between applied pressure and pore diameter.

Nitrogen adsorption–desorption measurements were performed at 77 K using a Quadrasorb EVO Surface Area & Pore Size Analyser (Quantachrome Instruments, Boynton Beach, FL, USA). Before analysis, the samples were degassed under vacuum at 150 °C for 3 h to remove physically adsorbed moisture. The specific surface area was calculated using the Brunauer–Emmett–Teller (BET) method, and pore-volume characteristics were derived from the adsorption data. Data acquisition and analysis were performed using ASiQwin™ software (version 3.01, Quantachrome Instruments).

#### 2.3.3. Scanning Electron Microscopy

The surface morphology and microstructural features of the diatomite samples were examined using a ZEISS Crossbeam 540 field-emission scanning electron microscope (Carl Zeiss Microscopy GmbH, Oberkochen, Germany). The samples were mounted on SEM holders using conductive adhesive tape and examined at an accelerating voltage of 5 kV. Images were acquired at magnifications selected to resolve the characteristic morphology and porous structure of the diatomite particles.

#### 2.3.4. X-Ray Diffraction Analysis

The mineralogical and crystalline phase composition of the untreated and modified diatomite samples was examined by powder X-ray diffraction using a SmartLab X-ray diffractometer (Rigaku Corporation, Tokyo, Japan) with Cu Kα radiation. Diffraction patterns were recorded in conventional θ/2θ geometry over a 2θ range of 5–70°, with a step size of 0.02°. The X-ray source was operated at 40 kV and 30 mA. The principal mineral phases were identified by matching the experimental diffraction patterns with reference patterns from the ICDD Powder Diffraction File (PDF) database, and changes in crystalline phase composition induced by acid treatment, calcination, and flux-assisted modification were subsequently evaluated.

### 2.4. Water-Extractable Ionic Components

Water-extractable ionic components were determined to evaluate the effect of the purification and modification procedures on the fraction of ionic species released from the diatomite into water under the specified extraction conditions. Aqueous extracts were prepared by mixing 30 g of each sample with 150 mL of deionized water. The mixtures were mechanically shaken for 5 min and subsequently filtered. The resulting filtrates were analyzed by ion chromatography using a Dionex ICS 6000/Aquion system (Thermo Fisher Scientific, Waltham, MA, USA).

Anions were separated by anion-exchange chromatography using a DionexIonPac AS11-HC analytical column (4 μm, 2 × 250 mm) with a carbonate/bicarbonate eluent (3.5 mM Na_2_CO_3_/1.0 mM NaHCO_3_) at a flow rate of 1.2 mL/min and detected by suppressed conductivity. Cations were separated by cation-exchange chromatography using a DionexIonPac CS12A analytical column (5 μm, 3 × 150 mm) with 20 mM methanesulfonic acid at a flow rate of 1.0 mL/min and detected by conductivity. Quantitative measurements were performed in five independent determinations (*n* = 5), and the results are reported as mean ± standard deviation (SD).

### 2.5. Antibacterial Evaluation

The antibacterial performance of the modified diatomite sorbent was evaluated using a laboratory isolate of *Enterococcus faecium* obtained by the authors from the root-associated microbiota of halophytic plants collected in the Karaganda Region, Kazakhstan. The bacterial isolate was identified as *E. faecium* by matrix-assisted laser desorption/ionization time-of-flight mass spectrometry (MALDI-TOF MS) using reference spectral database matching.

For the time-dependent contact experiment, three experimental conditions were evaluated: bacterial culture without sorbent (control), bacterial culture in contact with untreated natural diatomite (DE), and bacterial culture in contact with DE-H_2_SO_4_-600f. For the sorbent-containing groups, 1 mL of bacterial suspension was applied to1000 mg of sorbent. Bacterial abundance was evaluated at 0, 1, 3, and 7 h by measuring optical density at 600 nm (OD_600_) and by determining viable bacterial counts as colony-forming units per milliliter (CFU/mL). At each sampling time, aliquots were serially diluted and plated on LB agar. The plates were incubated at 37 °C for 24 h, after which colonies were counted and viable bacterial concentrations were expressed as CFU/mL. The measurements obtained at 0 h were used as the experimental baseline for each condition. The experiment was performed in two independent biological replicates, with three analytical replicates for each measurement.

In a separate experiment conducted at a high sorbent-to-liquid ratio, 0.25 mL of an *E. faecium* suspension containing approximately 1.8 × 10^7^ CFU/mL was applied to 50 g of DE-H_2_SO_4_-600f. The material was maintained in contact with the bacterial suspension for 1 h and subsequently allowed to dry for 1 h. The treated material was then transferred onto LB (Luria–Bertani) agar and incubated at 37 °C for 24 h. Bacterial growth was assessed after incubation.

### 2.6. Statistical Analysis

Quantitative XRF and ion-chromatography data are reported as mean ± standard deviation (SD) based on five independent measurements (*n* = 5). For the antibacterial evaluation, two independent biological replicates were performed, with three analytical replicates for each measurement. OD_600_ and CFU/mL values are reported as mean ± SD. Descriptive statistical analysis was applied to summarize the experimental data; no inferential statistical tests or *p*-values were used.

## 3. Results

### 3.1. Changes in Chemical Composition During Diatomite Modification

Natural diatomite contains mineral-associated components that can influence the composition and accessible structure of the material. Therefore, the first stage of the study evaluated changes in the chemical composition of natural diatomite produced by the applied purification and modification procedures.

The chemical compositions of untreated diatomite (DE) and the corresponding treated samples were compared by XRF. The contents of SiO_2_, Fe_2_O_3_, Al_2_O_3_, and TiO_2_ are presented in Figure 1.

Washing and deionization produced relatively small changes in the oxide composition, whereas acid treatment resulted in substantially greater changes in the Fe_2_O_3_, Al_2_O_3_, and TiO_2_ contents. Sulfuric-acid treatment produced the greatest reduction in these components among the investigated acid-treatment conditions. Compared with untreated diatomite, the Fe_2_O_3_, Al_2_O_3_, and TiO_2_ contents decreased by approximately 1.6-, 2.4-, and 1.3-fold, respectively. The decrease in these components was accompanied by an increase in the relative SiO_2_ content of the treated material, demonstrating enrichment of the silica-rich fraction during acid treatment.

### 3.2. Changes in Water-Extractable Ionic Components

Ion-chromatography analysis showed that washing and deionization produced a pronounced decrease in the concentrations of the measured water-extractable ionic components (Figure 2). For example, Na^+^ decreased from 92.20 to 5.08 mg/L, Ca^2+^ decreased from 27.55 to 3.71 mg/L, Cl^−^ decreased from 98.37 to 7.28 mg/L, and SO_4_^2−^ decreased from 78.81 to 2.97 mg/L.

Subsequent acid treatment produced comparatively small additional changes in this already reduced water-extractable fraction. Thus, the principal decrease in readily water-extractable ionic components occurred during washing and deionization rather than during the subsequent acid-treatment stage.

These results complement the XRF data: washing and deionization primarily removed readily water-extractable components, whereas acid treatment produced more pronounced changes in the solid-phase composition of the material.

### 3.3. Changes in Pore Structure and Surface Morphology

The porous structure of natural diatomite was first examined by mercury intrusion porosimetry. Untreated DE exhibited a broad pore-size distribution of approximately 20–5000 nm, with the largest effective pore volume associated with pores in the range of approximately 500 nm to 1.5 μm (Figure 3).

SEM examination confirmed the characteristic porous morphology of the natural diatomite particles and showed pore dimensions of approximately 0.8–1.3 μm (Figure 4). The SEM observations were therefore consistent with the principal pore-size range identified by mercury intrusion porosimetry.

Nitrogen-sorption analysis demonstrated substantial treatment-induced changes in the pore characteristics of the material (Figure 5). Deionization increased both the specific surface area and pore volume relative to untreated DE, and acid treatment produced a further increase. Sulfuric-acid treatment increased the specific surface area by approximately 51.5% relative to untreated DE. In contrast, calcination at 900 °C caused a pronounced decrease in both specific surface area and pore volume, indicating substantial restructuring of the porous material at the higher treatment temperature.

### 3.4. Changes in Crystalline Phase Composition

X-ray diffraction analysis was used to examine changes in the mineralogical and crystalline phase composition of diatomite produced by acid treatment, calcination, and flux-assisted modification (Figure 6). Untreated diatomite contained quartz and opal together with illite and plagioclase phases.

Following sulfuric-acid treatment and calcination at 600 °C, the contributions associated with illite and plagioclase decreased, while changes in the silica-containing phases were observed. Cristobalite reflections were detected in the thermally treated material. In DE-H_2_SO_4_-600f, the cristobalite-associated reflection near 22° 2θ became more pronounced, indicating an additional change in crystalline phase composition following Na_2_CO_3_-assisted calcination.

Taken together, the compositional, pore-structure, and phase-analysis results demonstrate that sequential sulfuric-acid treatment and calcination at 600 °C with Na_2_CO_3_ produced a substantially modified diatomite material. DE-H_2_SO_4_-600f combined a silica-enriched composition with altered pore characteristics and a modified crystalline phase composition and was therefore selected for subsequent evaluation of its interaction with *E. faecium*.

### 3.5. Interaction of Modified Diatomite with Enterococcus faecium

The interaction of DE-H_2_SO_4_-600f with *E. faecium* was evaluated over 7 h and compared with untreated diatomite (DE) and a bacterial culture without sorbent (Table 2). Both OD_600_ and recoverable viable bacterial counts were monitored to characterize the bacterial response during contact with the sorbent.

In the control culture, bacterial growth was evident from the increase in both OD_600_ and viable counts, with CFU/mL increasing from 1.7 × 10^7^ at 0 h to 2.9 × 10^9^ after 7 h. In the presence of untreated DE, recoverable viable counts remained close to the initial level throughout the experiment, reaching 1.1 × 10^7^ CFU/mL after 7 h.

A markedly different response was observed with DE-H_2_SO_4_-600f. Recoverable viable counts decreased from 1.2 × 10^7^ CFU/mL at 0 h to 2.0 × 10^6^ CFU/mL after 1 h and to 1.0 × 10^4^ CFU/mL after 7 h. These values correspond to approximately 6-fold and 1200-fold reductions, respectively, relative to the initial recoverable viable count for this group. The accompanying decrease in OD_600_ was consistent with the pronounced change observed in viable bacterial recovery.

Thus, DE-H_2_SO_4_-600f showed a substantially stronger effect on recoverable viable *E. faecium* than untreated natural diatomite under the tested contact conditions. The CFU data establish a pronounced reduction in recoverable viable bacteria; the extent to which this reduction reflects bacterial inactivation versus adsorption or immobilization within the porous sorbent is considered separately in the Discussion.

### 3.6. Antibacterial Performance Under High Sorbent-to-Liquid Ratio Conditions

A separate experiment was performed to evaluate the interaction of DE-H_2_SO_4_-600f with *E. faecium* under conditions of a high sorbent-to-liquid ratio. A 0.25 mL bacterial suspension containing approximately 1.8 × 10^7^ CFU/mL was applied to 50 g of DE-H_2_SO_4_-600f. After 1 h of contact followed by 1 h of drying, the treated material was transferred onto LB agar and incubated at 37 °C for 24 h.

No bacterial growth was detected after incubation under the tested conditions. This result demonstrates that, at the high sorbent-to-liquid ratio used in this experiment, recoverable bacterial growth was suppressed below the level detectable by the applied cultivation procedure.

The result complements the time-dependent experiment presented in Section 3.5, in which DE-H_2_SO_4_-600f produced a progressive decrease in recoverable viable *E. faecium* counts over 7 h. However, because the present experiment assessed subsequent bacterial growth after contact with the sorbent rather than direct recovery of cells from the porous material, the absence of detectable growth should not be interpreted as evidence of complete bacterial killing.

## 4. Discussion

The results demonstrate that the successive treatment stages performed different but complementary functions in transforming natural diatomite into the final DE-H_2_SO_4_-600f material. Washing and deionization primarily removed readily water-extractable ionic components, whereas acid treatment produced more pronounced changes in the solid-phase composition and porous structure. Subsequent thermal and flux-assisted treatment further altered the pore characteristics and crystalline phase composition. Thus, the properties of DE-H_2_SO_4_-600f resulted from the combined effect of the sequential modification procedure rather than from a single treatment step.

Acid treatment produced a clear enrichment of the silica fraction, accompanied by a decrease in several mineral-associated components. This is consistent with previous and recent studies showing that acid treatment of diatomite selectively removes mineral impurities, enriches the silica fraction, and modifies the accessible surface area and porous structure [8,9,30,31]. In the present study, these compositional changes were accompanied by an increase in specific surface area, with H_2_SO_4_ treatment producing an approximately 51.5% increase relative to untreated DE. The agreement between the compositional and nitrogen-sorption data indicates that removal of acid-soluble mineral components was associated with greater accessibility of the porous silica framework.

The analysis of water-extractable ions reveals a different aspect of the purification process. Concentrations of Na^+^, Ca^2+^, Cl^−^, SO_4_^2−^, and the other measured ionic components decreased sharply after washing and deionization, whereas subsequent acid treatment produced comparatively small additional changes. This distinction is important because the XRF and ion-chromatography results describe different fractions of the material. Washing predominantly removed readily water-extractable species, while acid treatment produced more substantial changes in the solid-phase composition. Together, these results show why both stages were retained in the modification sequence.

Thermal treatment introduced an additional structural effect. Calcination at 900 °C resulted in a pronounced decrease in specific surface area and pore volume, showing that higher-temperature treatment did not simply improve the porous characteristics of the material. Thermal restructuring and phase transformations of diatomite during calcination have also been reported previously [7,32]. In the present study, treatment at 600 °C preserved more favorable pore characteristics while producing measurable changes in crystalline phase composition.

The addition of Na_2_CO_3_ during calcination further altered the XRD pattern of the sulfuric-acid-treated material. In DE-H_2_SO_4_-600f, the cristobalite-associated reflection near 22° 2θ became more pronounced, demonstrating that flux-assisted treatment affected the crystalline silica phases. Flux-assisted thermal treatment has previously been shown to promote physicochemical and phase changes in diatomite [33]. Importantly, the present XRD results establish a change in phase composition; they do not by themselves demonstrate improved mechanical strength or stability of the resulting material.

The detection of a cristobalite-associated reflection also requires consideration in the context of the intended respiratory application. Cristobalite is a crystalline silica phase, and health concerns are specifically associated with inhalation exposure to respirable crystalline silica particles [34]. The present XRD analysis establishes the presence of a cristobalite-associated crystalline phase but does not determine whether respirable particles would be released from the sorbent during use. Therefore, particle-release and inhalation-exposure testing will be required before DE-H_2_SO_4_-600f can be incorporated into a finished face-mask product.

The physicochemical changes were accompanied by a pronounced difference in the interaction of the modified material with *E. faecium*. In the untreated-DE group, recoverable viable counts remained close to the initial level during the 7 h experiment. In contrast, DE-H_2_SO_4_-600f reduced recoverable viable counts from 1.2 × 10^7^ CFU/mL to 2.0 × 10^6^ CFU/mL after 1 h and to 1.0 × 10^4^ CFU/mL after 7 h, corresponding to approximately 6-fold and 1200-fold reductions, respectively. The magnitude and progressive nature of this difference demonstrate that the biological behavior of the modified material cannot be attributed simply to the presence of natural diatomite.

At the same time, the CFU results should be interpreted according to what was directly measured. Diatomite is a highly porous adsorbent, and both earlier and recent studies have shown that bacterial cells can be retained or immobilized on diatomite-based carriers and that modification of the diatomite surface can substantially influence bacteria–material interactions [19,20,21]. Consequently, the reduction in recoverable CFU observed here does not by itself distinguish bacterial inactivation from adsorption or immobilization within DE-H_2_SO_4_-600f. Importantly, this mechanistic limitation does not alter the magnitude of the observed effect: after 7 h of contact, recoverable viable bacterial counts were approximately 1200-fold lower than the initial level. Direct recovery and viability measurements would be required to determine the relative contributions of bacterial retention and inactivation.

Other antibacterial diatomite-based materials reported in the literature commonly employ additional active components, including biologically active compounds [22] or silver-containing phases [23], whereas no such antibacterial agent was introduced into DE-H_2_SO_4_-600f. The pronounced reduction in recoverable viable bacteria observed here was therefore achieved following physicochemical modification of the diatomite itself.

The separate experiment performed at a high sorbent-to-liquid ratio provides complementary evidence. No bacterial growth was detected after 24 h under the tested conditions following contact of the bacterial suspension with DE-H_2_SO_4_-600f and subsequent incubation of the material on LB agar. Because this experiment used a different exposure configuration, it should not be treated as a quantitative extension of the 7 h CFU experiment. Rather, the two experiments demonstrate the behavior of the material under two different contact conditions: a progressive reduction in recoverable viable bacteria in the time-dependent experiment and absence of detectable subsequent growth under the high sorbent-to-liquid ratio conditions.

These findings are relevant to the intended use of DE-H_2_SO_4_-600f as a functional sorbent component in medical face masks. The material is not proposed as a replacement for the conventional filtration layer. Instead, its role would be to provide an additional porous sorptive component capable of taking up deposited liquid and retaining associated biological contaminants [4,35]. Translation from the sorbent material to a finished multilayer mask will require engineering and validation of the filter configuration, including measuring the filtration efficiency, pressure drop, breathability, particle retention and shedding, and biological performance under aerosol exposure. A schematic representation of the proposed incorporation of the modified diatomite sorbent into a multilayer medical face mask is shown in Figure 7.

Recent evaluations of antibacterial face masks similarly emphasize that antibacterial performance should be considered together with conventional filtration characteristics when assessing the functionality of the finished product [29].

The sorptive characteristics of modified diatomite may also be relevant to protective applications in industrial environments where inhalation exposure involves contaminant-bearing dust and aerosol particles. Natural and modified forms of diatomite have been investigated for the sorption of radionuclides and radiometals, including Th(IV), U(VI), ^60^Co(II), and ^137^Cs [10,11,12,13]. Recent studies continue to demonstrate interest in modified diatomite and advanced sorbent systems for uranium and thorium removal [14,15], while the environmental and occupational relevance of contamination associated with thorium-bearing materials has also been highlighted [16]. In environments associated with the mining and processing of thorium- and uranium-bearing materials, incorporation of a sorptive mineral component into respiratory protective systems may therefore provide an additional means of capturing and retaining contaminant-bearing particles. This function should be distinguished from radiation shielding: the proposed role of the diatomite layer is the sorption and retention of aerosol-borne contaminants rather than attenuation of ionizing radiation.

For incorporation into a finished respiratory protective product, the sorbent would need to be securely immobilized within the multilayer structure, with particle release and shedding evaluated under realistic airflow and mechanical conditions. Thus, the present results establish the physicochemical properties of the modified sorbent and demonstrate a pronounced reduction in recoverable viable bacteria, while its integration into a finished respiratory protective system represents the next stage of development.

## 5. Conclusions

Sequential physicochemical modification of natural diatomite from the Zhalpak deposit produced a silica-enriched porous sorbent with substantially altered physicochemical characteristics and a marked decrease in recoverable bacterial counts under the tested conditions. The successive treatment stages had distinct but complementary effects on the material. Washing and deionization primarily removed readily water-extractable ionic components, whereas acid treatment produced more substantial changes in the solid-phase composition and increased the accessibility of the porous silica framework. H_2_SO_4_ treatment increased the specific surface area by approximately 51.5% relative to untreated diatomite. Subsequent thermal and Na_2_CO_3_-assisted treatment further modified the pore characteristics and crystalline phase composition, leading to the final DE-H_2_SO_4_-600f sorbent. The combined compositional, surface-area, pore-structure, morphological, and phase-characterization results demonstrate that the properties of the final material arose from the cumulative effects of sequential physicochemical modification rather than from a single treatment step.

The modification also produced a pronounced change in the interaction of diatomite with *Enterococcus faecium*. Aftercontact with DE-H_2_SO_4_-600f, recoverable viable bacterial counts decreased from 1.2 × 10^7^ CFU/mL to 2.0 × 10^6^ CFU/mL after 1 h and to 1.0 × 10^4^ CFU/mL after 7 h, corresponding to approximately 6-fold and 1200-fold reductions, respectively. In contrast, bacterial counts in the presence of untreated diatomite remained at approximately 10^7^ CFU/mL over the same period, while substantial bacterial growth occurred in the control. In the separate high sorbent-to-liquid ratio experiment, no bacterial growth was detected after 24 h under the tested conditions.

An important outcome of this study is the combination of tailored porous and sorptive characteristics with a marked effect on bacterial recovery within a single modified diatomite material. Unlike antibacterial diatomite-based materials that rely on additional biologically active or silver-containing components, this effect was observed for DE-H_2_SO_4_-600f following physicochemical modification of the diatomite itself, without the addition of such components. This finding expands the functional potential of natural diatomite beyond its conventional role as a porous sorbent and demonstrates the value of sequential modification for developing mineral-based materials with combined sorptive and antibacterial properties.

These characteristics support the potential future incorporation of DE-H_2_SO_4_-600f as a functional sorbent component in multilayer medical face masks, where a porous mineral layer could complement conventional filtration materials by contributing additional sorptive capacity and retention of deposited contaminants. More broadly, modified diatomite may also be considered for respiratory-protection systems intended for environments containing contaminant-bearing dust and aerosol particles, including those associated with the mining and processing of thorium- and uranium-bearing materials.

## Figures and Tables

**Figure 1:**
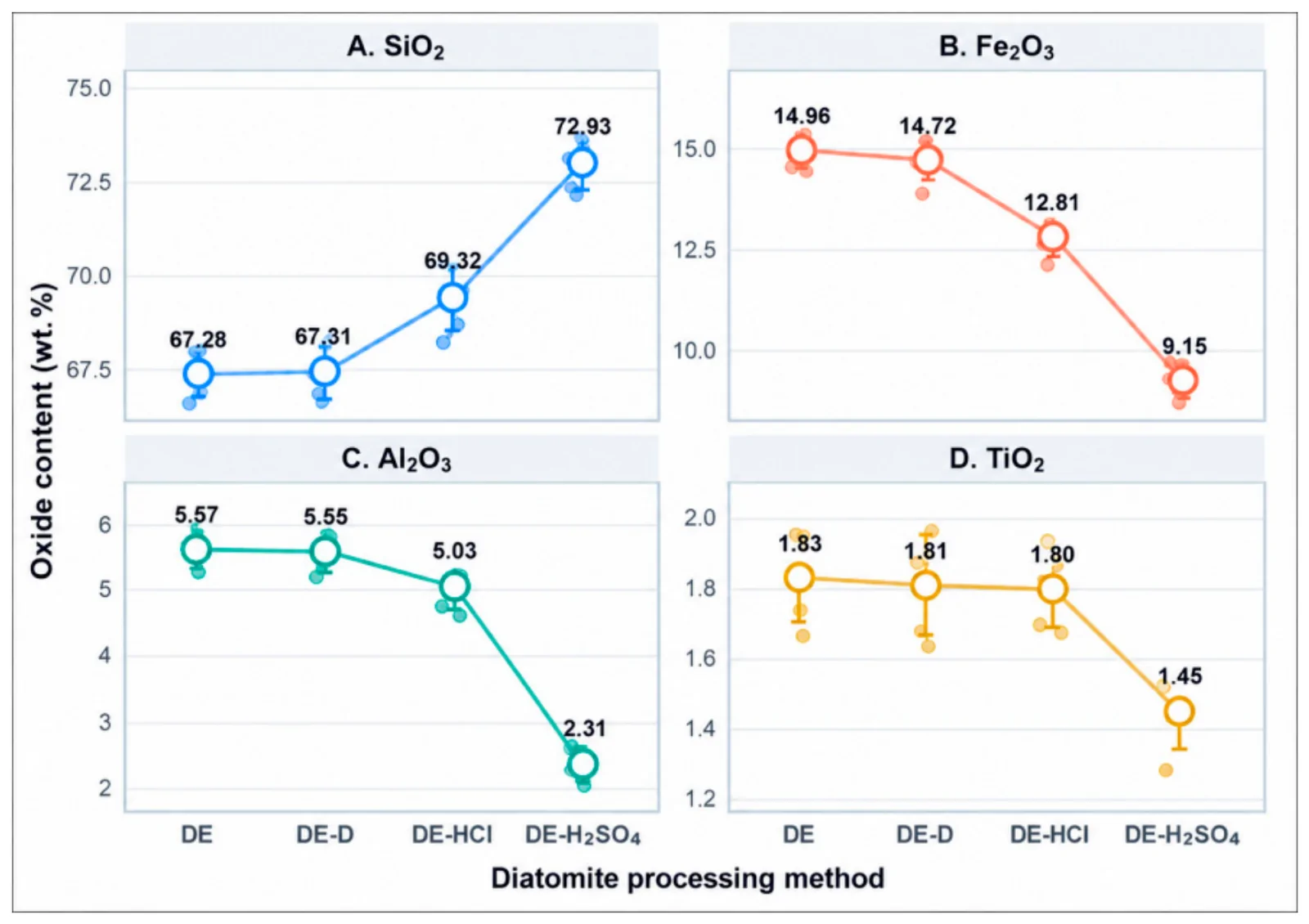
Effect of different processing methods on the selected oxide composition of diatomite-based sorbents. The contents of SiO_2_, Fe_2_O_3_, Al_2_O_3_, and TiO_2_ are expressed as wt.%. Values are presented as mean ± SD (*n* = 5).

**Figure 2:**
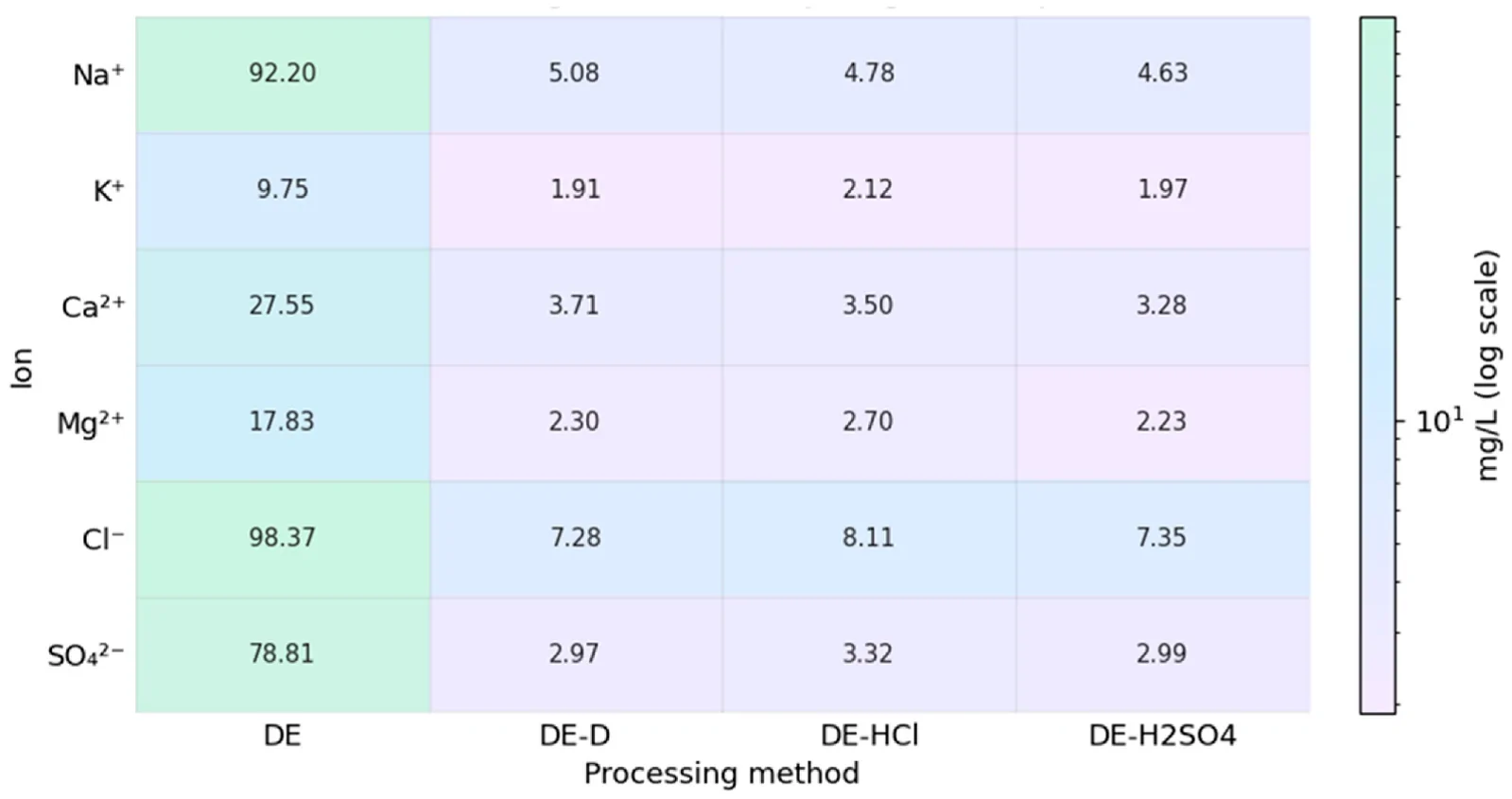
Effect of diatomite treatment on the concentrations of water-extractable ionic components in aqueous extracts. Values are presented as mean ± SD (*n* = 5).

**Figure 3:**
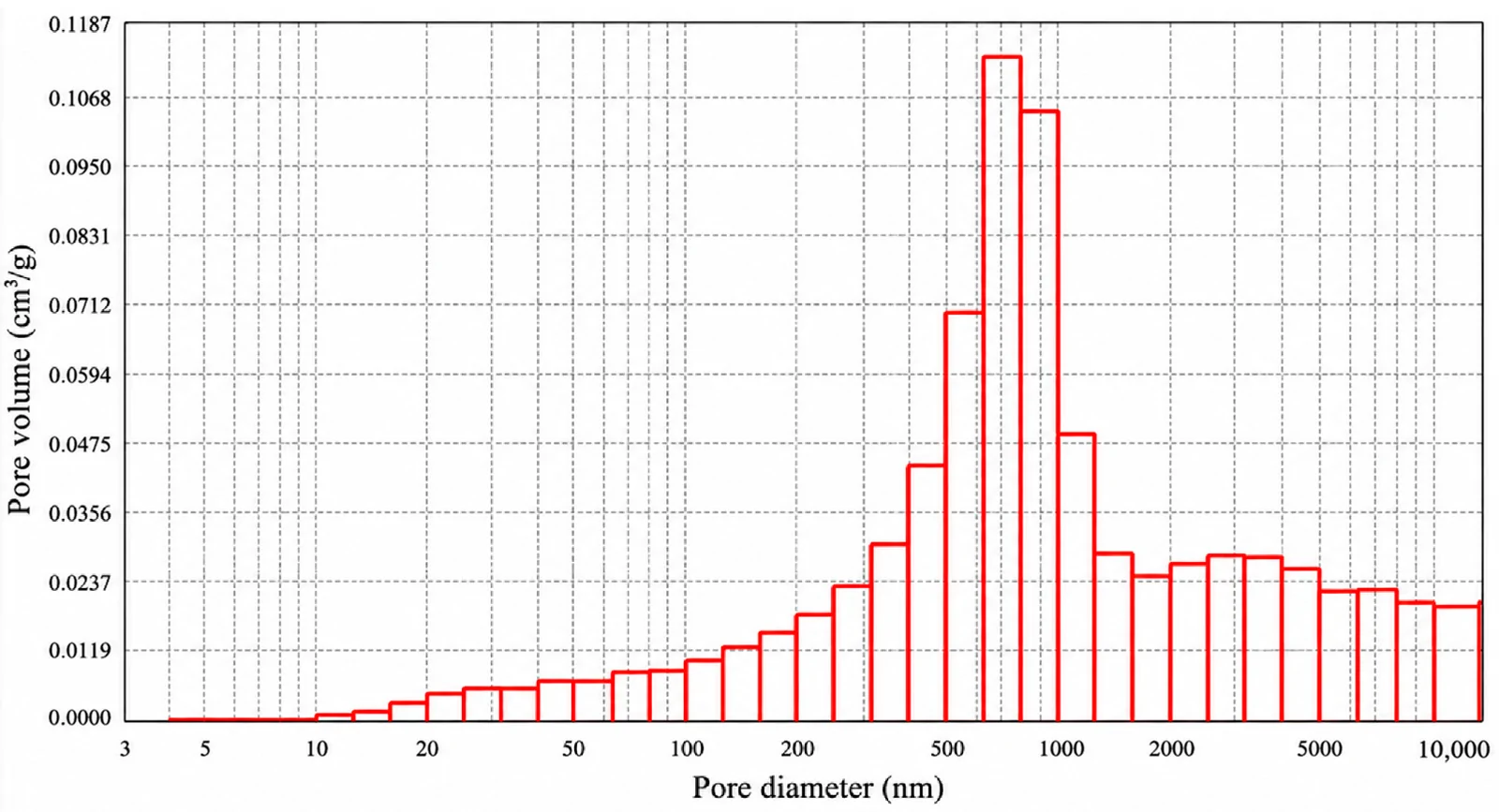
Pore-size distribution of untreated natural diatomite (DE) determined by mercury intrusion porosimetry.

**Figure 4:**
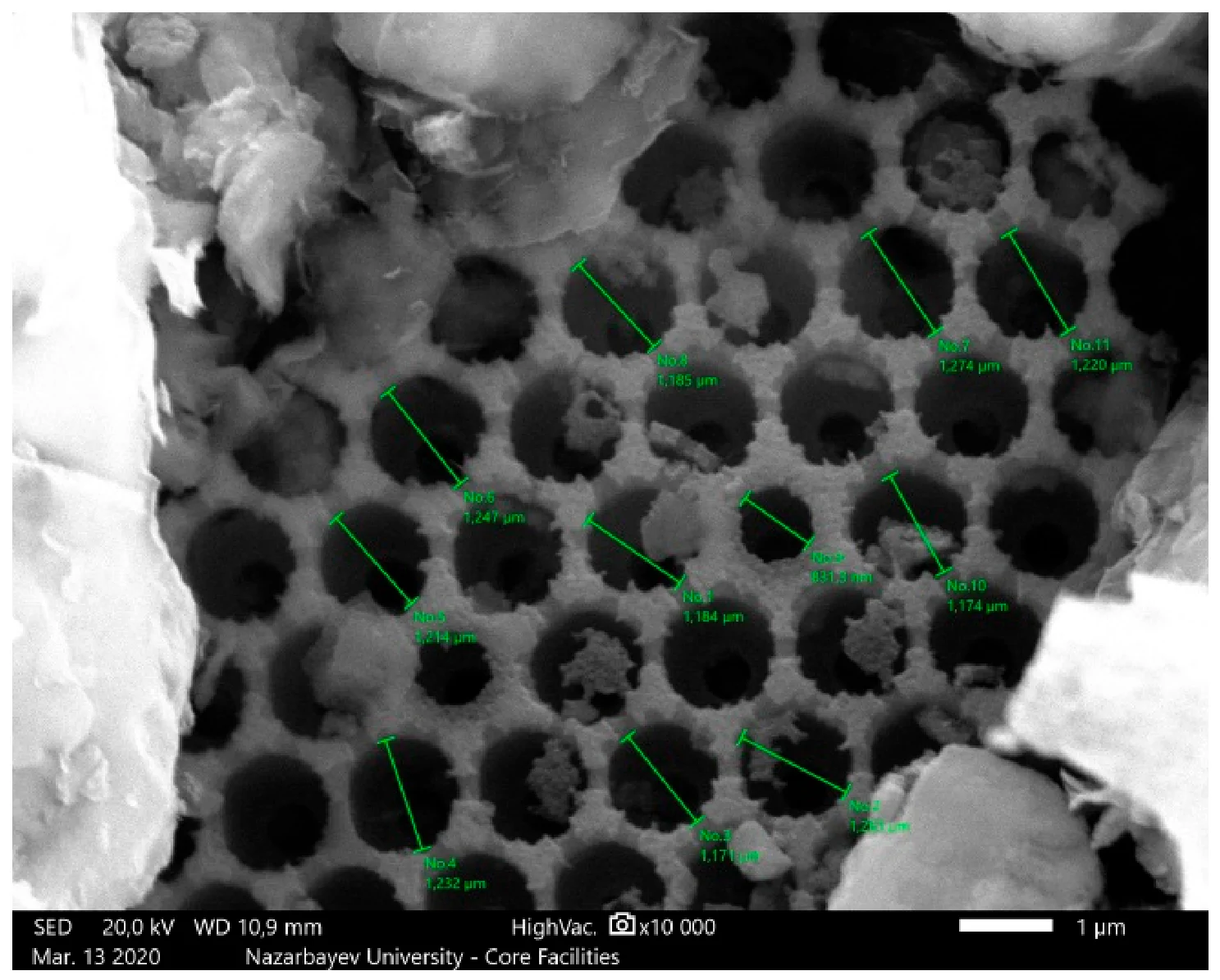
SEM image showing the characteristic porous morphology of untreated natural diatomite (DE).

**Figure 5:**
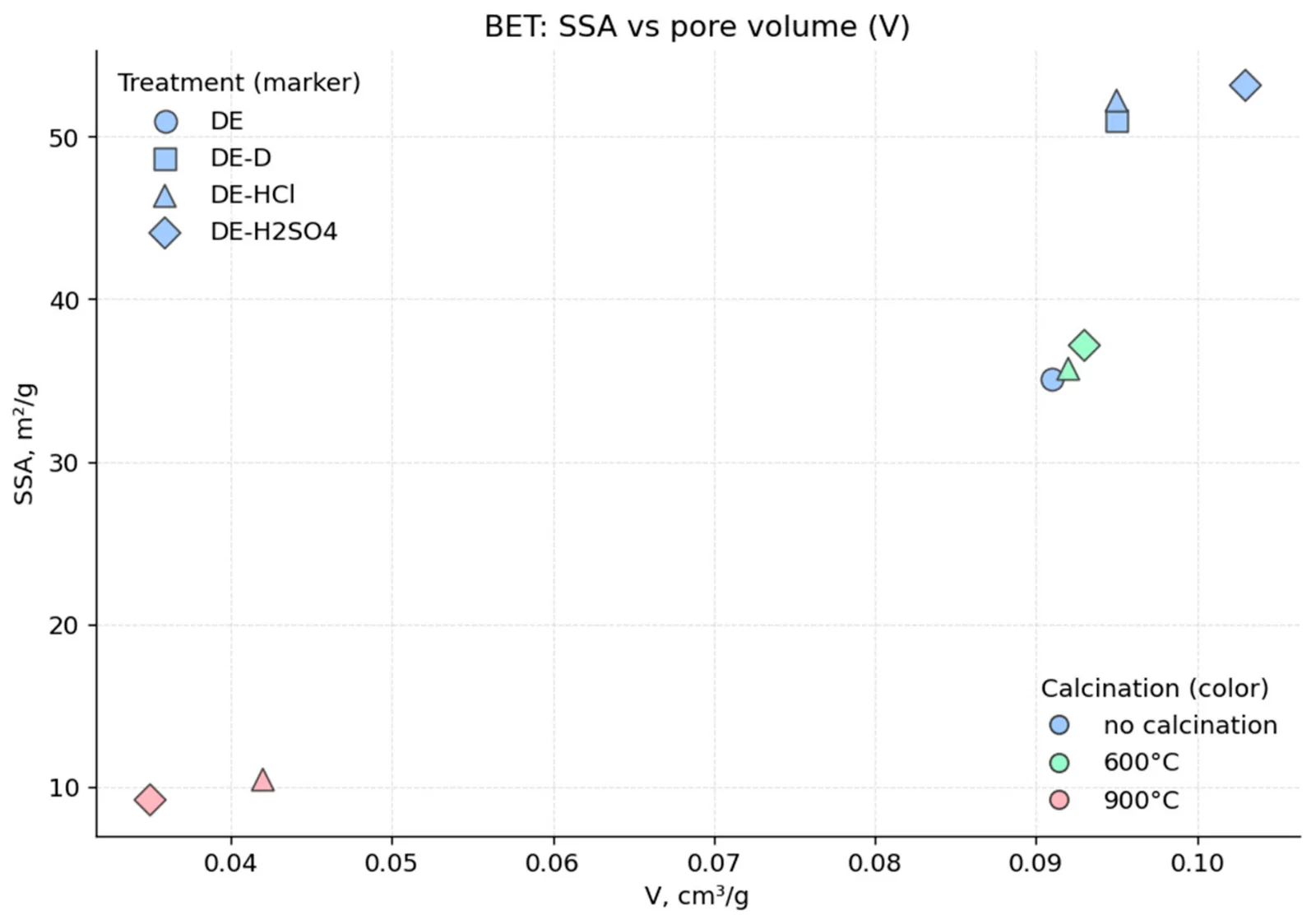
Effect of purification, acid treatment, and calcination on the specific surface area and pore volume of diatomite samples.

**Figure 6:**
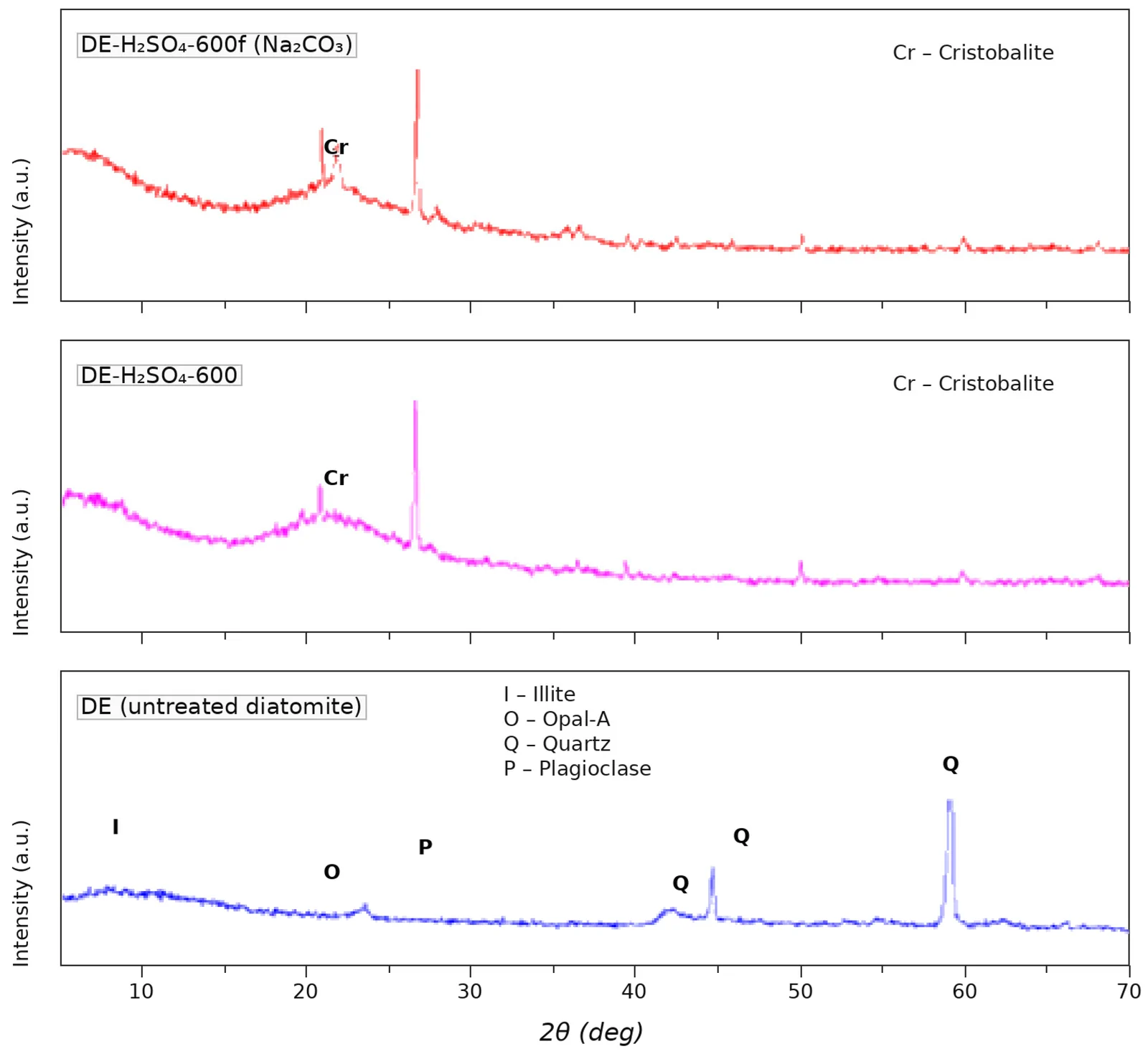
X-ray diffraction patterns of untreated and modified diatomite samples showing changes in crystalline phase composition following acid treatment, calcination, and Na_2_CO_3_-assisted modification.

**Figure 7:**
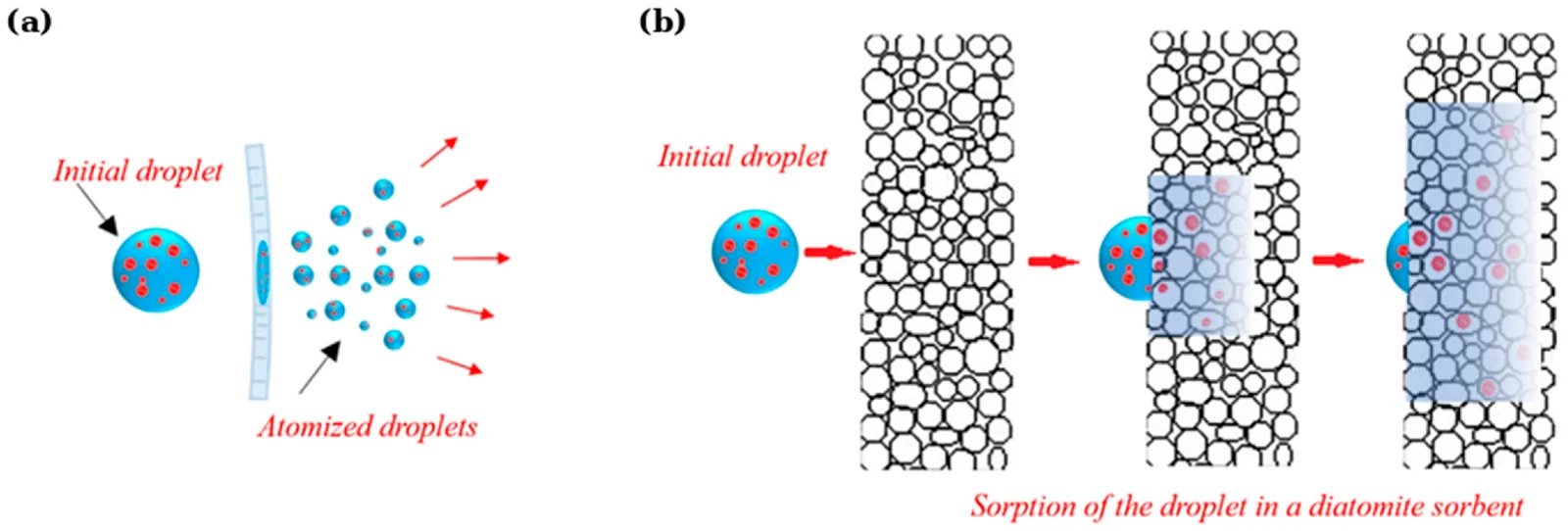
Schematic representation of the proposed incorporation of the modified diatomite sorbent into a multilayer medical face mask: (**a**) atomization of the initial droplet into smaller droplets; (**b**) uptake of the atomized droplets by the diatomite sorbent. The diatomite-containing layer is intended to provide an additional porous sorptive component for the uptake of deposited droplets and retention of associated contaminants, while the conventional filter layers provide the primary filtration function.

**Table 1:** Chemical composition of natural diatomite from the Zhalpak deposit.

Diatomite Composition	SiO_2_	Al_2_O_3_	Fe_2_O_3_	K_2_O	MgO	Na_2_O	TiO_2_	CaO	MnO	P_2_O_5_	SO_3_	Others
Content, %	67.34	15.51	2.90	1.52	2.22	1.43	0.69	0.79	0.02	0.16	0.48	6.94

**Table 2:** Effect of untreated diatomite (DE) and DE-H_2_SO_4_-600f on OD_600_ and recoverable viable *E. faecium* counts during 7 h of contact.

Time (h)	Control OD_600_	Control CFU/mL	DE OD_600_	DE CFU/mL	DE-H_2_SO_4_-600f OD_600_	DE-H_2_SO_4_-600f CFU/mL
0	0.4 ± 0.1	(1.7 ± 0.07) × 10^7^	0.4 ± 0.1	(1.7 ± 0.07) × 10^7^	0.2 ± 0.1	(1.2 ± 0.08) × 10^7^
1	1.2 ± 0.07	(2.0 ± 0.07) × 10^8^	0.3 ± 0.1	(1.3 ± 0.07) × 10^7^	0.12 ± 0.1	(2.0 ± 0.08) × 10^6^
3	1.74 ± 0.06	(2.5 ± 0.07) × 10^9^	0.2 ± 0.1	(1.1 ± 0.07) × 10^7^	0.09 ± 0.1	(1.2 ± 0.08) × 10^4^
7	1.74 ± 0.06	(2.9 ± 0.07) × 10^9^	0.2 ± 0.1	(1.1 ± 0.07) × 10^7^	0.05 ± 0.1	(1.0 ± 0.08) × 10^4^

**Note:** Values are presented as mean ± SD. CFU, colony-forming units; DE, untreated natural diatomite; DE-H_2_SO_4_-600f, sulfuric-acid-treated diatomite calcined at 600 °C with Na_2_CO_3_ flux.

## Data Availability

The original contributions presented in this study are included in the article. Further inquiries can be directed to the corresponding author.
